# Severe alpha-1 antitrypsin deficiency in composite heterozygotes inheriting a new splicing mutation QO_Madrid_

**DOI:** 10.1186/s12931-014-0125-y

**Published:** 2014-10-07

**Authors:** Beatriz Lara, Maria Teresa Martínez, Ignacio Blanco, Cristina Hernández-Moro, Eladio A Velasco, Ilaria Ferrarotti, Francisco Rodriguez-Frias, Laura Perez, Irene Vazquez, Javier Alonso, Manuel Posada, Beatriz Martínez-Delgado

**Affiliations:** Servicio de Neumología, Hospital Universitario Arnau de Vilanova, Lleida, Spain; Servicio de Neumología, Hospital Universitario Doce de Octubre, Madrid, Spain; Board of Directors of the Alpha-1 Antitrypsin Deficiency Spanish Registry, Lung Foundation Breathe, Spanish Society of Pneumology (SEPAR), Barcelona, Spain; Grupo de Splicing y Cáncer, Instituto de Biología y Genética Molecular (CSIC-UVa), Valladolid, Spain; Center for Diagnosis of Inherited Alpha-1 Antitrypsin Deficiency, Department of Molecular Medicine, Section of Pneumology, IRCCS San Matteo Hospital Foundation, University of Pavia, Pavia, Italy; Servicio de Bioquímica, Hospital Universitario Vall d’ Hebron, Barcelona, Spain; Molecular Genetics Unit, Instituto de Investigación en Enfermedades Raras (IIER), Instituto de Salud Carlos III (ISCIII), Carretera Majadahonda-Pozuelo Km 2,200, Majadahonda, Madrid, 28220 Spain; Human Genetics Area, Instituto de Investigación en Enfermedades Raras (IIER), Instituto de Salud Carlos III (ISCIII), Madrid, Spain; Instituto de Investigación en Enfermedades Raras (IIER), Instituto de Salud Carlos III (ISCIII), Spain RDR and CIBERER, Madrid, Spain

**Keywords:** Alpha-1 antitrypsin, Allelic variants, Null alleles, QO alleles, Splicing, Minigenes

## Abstract

**Background:**

Severe Alpha-1 Antitrypsin (AAT) deficiency is a hereditary condition caused by mutations in the *SERPINA1* gene, which predisposes to lung emphysema and liver disease. It is usually related to PI*Z alleles, and less frequent to rare and null (QO) alleles. Null-AAT alleles represent the end of a continuum of variants associated with profound AAT deficiency and extremely increased risk of emphysema.

**Methods:**

A family with severe AAT deficiency was analyzed to achieve genetic diagnosis. The complete exons and introns of the *SERPINA1* gene were sequenced and transcriptional analysis by RT-PCR was performed to characterize the effect of splicing variants found in the patients. In addition, a minigene MGserpa1_ex1b-1c was cloned into the pSAD vector to in vitro investigate the independent impact of variants on splicing process.

**Results:**

We report a new identified null allele (PI*QO_Madrid_) in two adult siblings with practically no detectable serum AAT. The PI*QO_Madrid_ allele consist of a duplication of the thymine (T) in position +2 of the donor splice site of exon 1C (+2dupT). In these two subjects, PI*QO_Madrid_ occurred in compound heterozygote combination with the previously described variant PI*QO_Porto_. Both QO_Madrid_ and QO_Porto_ variants are located very close together in a regulatory region of the *SERPINA1* gene. Analysis of transcripts revealed that QO_Madrid_ variant prevented the expression of transcripts from exon 1C, and then normally spliced RNA products are not expected in the liver of these patients. In addition, aberrant splicing patterns of both variants were clearly distinguished and quantified by functional in vitro assays lending further support to their pathogenicity.

**Conclusion:**

Finding pathogenic mutations in non-coding regions of the *SERPINA1* highlight the importance that regulatory regions might have in the disease. Regulatory regions should be seriously considered in discordant cases with severe AAT deficiency where no coding mutations were found.

**Electronic supplementary material:**

The online version of this article (doi:10.1186/s12931-014-0125-y) contains supplementary material, which is available to authorized users.

## Background

Human alpha-1 antitrypsin (AAT), also named alpha-1 protease inhibitor (α1-PI) and SERPINA1 (Serine Protease Inhibitor, group A, member 1), is a circulating glycoprotein with a broad spectrum antiserine-protease activity, including the inhibition of free elastase from neutrophil. AAT acts mainly as an acute phase reactant but also has anti-inflammatory, anti-infectious and immunomodulation effects [1,2]. Deficiency of AAT leads to lung tissue damage and emphysema due to uncontrolled elastase activity, or liver disease caused by accumulation within the hepatocytes of misfolded, aggregated AAT protein [3].

Severe AAT deficiency is an inherited condition characterized by AAT serum levels below 35% (or 50 mg/dL) the normal value. The protein is codified at the protease inhibitor locus (14q32.1), by the *SERPINA1* gene, which is organized into three non-coding exons (1A, 1B and 1C) and four coding exons (2–5) [4]. The *SERPINA1* gene is inherited following an autosomal co-dominant pattern [5].

Over 80% of AAT is synthesized in the liver, although other cell types such as blood monocytes, neutrophils, macrophages, pancreas, endothelium, enterocytes, lung alveolar and some cancer cells are also capable of secreting additional quantities [6,7]. Transcriptional regulation occurs in at least two different sites within the gene: the hepatocyte promoter located upstream the transcription start site of exon 1C, and the monocyte promoter located upstream of exon 1A [8].

More than one hundred different genetic variants have been already described in detail and a wide knowledge about how these alterations affect the protein conformation and function has been learnt in the last decades [9]. Allelic variants of AAT are conventionally classified as normal and deficient [1,3,5,10]. The most common normal AAT alleles are PI*M1 (rs6647; NM_000295.4:c.710 T > C; p.Val213Ala, mature protein), PI*M2 (rs709932; c.374G > A; p.Arg101His) and PI*M3 (rs1303; c.1200A > C; p.Glu376Asp). The allelic frequency of these polymorphisms varies among populations being M1 (24%), M2 (13%), and M3 (23%) (minor allele frequency source:1000 Genomes). In contrast to normal AAT alleles, there are two categories of genetic variants that cause AAT deficiency: the deficient and the null alleles. The most common deficient alleles are PI*S (rs17580: c. 863A > T; p. Glu264Val) and PI*Z (rs28929474; c.1096G > A; p.Glu342Lys), with a PI*S prevalence in Caucasians of 5-10% [11] (3%, 1000 Genomes) and a PI*Z prevalence of 1-3% (0.7%, 1000 Genomes). Normal serum levels are associated with M alleles. In contrast reduced levels are associated with the PI*S and PI*Z alleles with AAT serum levels of 40% and 10-20%, respectively. Then, the PI*Z allele is related to severe deficiency and is the phenotype most often associated with the disease.

There are also other rare variants, with a lower frequency ranging from 1 × 10^−4^ to 2.5 × 10^−5^, and around 15% serum AAT [10,12]. Both PI*S and PI*Z, and rare deficiency alleles M_Malton_, M_Duarte_, and S_Iiyama_ produce misfolded proteins which are retained into hepatocytes forming polymers, which can cause cell stress and liver damage, and on the other hand, as a result of polymerization/retention into hepatocytes, reduced blood and tissues concentration of AAT, insufficient to protect tissues against proteases [9,13].

In the last two decades about 25 null variants, associated with trace amounts (<1%) of plasma AAT, have been discovered (Table 1) [4,10,12,14-26]. Although little information about their prevalence is available, it is thought that they are extremely infrequent, with an estimated combined frequency of 1.4 × 10^−4^ among Caucasians. In fact, the few published cases of carriers of null alleles have been found on European and European-American individuals, with only three carriers found in descendants of Egyptians, African-Americans and Chinese. Notably, despite their low prevalence, null variants have a strong effect on phenotype, conferring an extraordinary risk to develop severe pulmonary emphysema [19,27,28]. The majority of these variants cause premature stop codons in the *SERPINA1* gene leading to either unstable mRNA or truncated, unstable proteins (i.e. QO_Granite Falls_, QO_Mattawa_, QO_Hong Kong_, QO_Bellingham_). Other mechanisms include complete gene deletion (QO_Isola di Procida_), missense mutations which probably destabilize the AAT protein (QO_Ludwigshafen_ and QO_New Hope_), or mutations affecting the RNA splicing process (QO_West_, QO_Bonny Blue_, QO_Porto_) [4,29]. Although infrequent, AAT null variants have allowed a better understanding of the molecular basis of the disease and revealed functionally critical regions of the gene sequence [27].

**Table 1:** Molecular and clinical features of known 21 PI*QO (Null) alleles

**Allele**	**Molecular basis of disease**	**Clinical features**	**Reference**
QO_amersfoort_	Exon 2. Tyr160 stop. Nonsense mutation producing a stop at codon 160, and a premature termination in exon 2 with no detectable mRNA	The index patient was a Caucasian 47-year-old female patient who had COPD	[30]
QO_bellingham_	Exon 3. Lys217 stop codon.	High risk of emphysema in homozygotes and compound heterozygotes	[31]
	No detectable AAT mRNA		
QO_bolton_	Exon 5. Δ1bpPro362 causing stop codon at 373. Truncated protein, degraded, not secreted	High risk of emphysema in homozygotes and compound heterozygotes	[17]
QO_bonny blue_	ΔG deletion position #1 of intron II splice acceptor	High risk of emphysema in homozygotes and compound heterozygotes	[4]
QO_cairo_	Exon 3. Lys259 stop codon. Truncated protein, degraded, not secreted protein.	High risk of emphysema in homozygotes. One carrier belonged to an Italian/Egyptian family, and 2 other to a family from Southern Italy	[26]
QO_clayton_	Exon 5. Pro362 insC causing stop codon at 376. Truncated protein, degraded and not secreted	High risk of emphysema in homozygotes and compound heterozygotes	[14]
QO_devon_ (=QO_newport_)	Exon 2. Gly115Ser. Intracellular degradation and reduced serum concentration	Risk of emphysema and liver disease in compound heterozygotes with Z allele. Unclear whether Gly115Ser would cause disease in absence of Z mutation	[32]
QO_granite falls_	Exon 2. Δ1bpTyr160 causing stop codon. No detectable AAT mRNA	Severe emphysema reported in an American black family Z compound heterozygote	[21]
QO_hong Kong_	Exon 4. Δ2bpLeu318 causing stop codon at 334. Truncated protein; degraded and not secreted	High risk of emphysema in homozygotes and compound heterozygotes. Reported in Chinese descents.	[24]
QO_isola di procida_	Δ17 Kb inc. exons II –V. No detectable AAT mRNA	Emphysema reported in compound heterozygote	[33]
QO_lisbon_	Exon 2. Thr68Ile.	High risk of emphysema in homozygotes.	[16]
		50% normal serum AAT in M/QO_Lisbon_ heterozygotes	
QO_ludwisghafen_	Exon 2. Ile92Asn. Intracellular degradation and no secreted protein	High risk of emphysema in homozygotes and compound heterozygotes	[18]
QO_madrid_	Intron 1C, c.-5 + 2dupT. Duplication of thymine in position +2 of the donor splice site, causing no expression of mRNA transcripts	Index case: a compound heterozygote QO_madrid_ /QO_porto_ with COPD. Three heterozygote siblings with radiological findings of lung disease.	[current report]
QO_mattawa_ (M1allele) and QO_ourém_ (M3 allele)	Exon 5. Same mutation Leu353Phe causing stop codon at 376, in M1 and M3 respectively. Truncated protein, degraded, not secreted, reduced serum levels	Lung emphysema reported in homozygotes	[15,25,29]
QO_milano_	Exon 3, 17 bp deletion (AAA CTA CAG CAC CTG GA), causing a stop codon downstream. Truncated protein lacking of active site	Heterozygote M/QO_milano_ Italian child with persistently increased in liver enzymes and a mild decrease in serum AAT levels	[23]
QO_new hope_	Exon 4, 5. Gly 320 GGG → Glu GAG/Glu 342 GAG → Lys AAG	High risk of emphysema in homozygotes and compound heterozygotes	[4]
QO_porto_	Intron 1C, c.-5 + 1G > A. Splicing site variant, causing no expression of mRNA transcripts.	High risk of emphysema in homozygotes.	[29]
QO_riedenburg_	Whole gene deletion. No AAT gene expression	High risk of emphysema in homozygotes and compound heterozygotes	[22]
QO_saarbueken_	Exon 5. 1158dupC causing stop codon at 376. Truncated protein; not secreted	High risk of emphysema in homozygotes.	[16]
		50% normal serum AAT in M/QO _Saarbueken_ heterozygotes	
QO_soest_	Exon 2. Thr102delA, which produces a TGA stop signal at codon 112	Index case: a homozygote 46-year-old man with severe COPD	[30]
QO_trastevere_	Exon 3. Try194 stop codon. Intracellular degradation of truncated protein; not secreted	Emphysema reported in an Italian compound heterozygote	[20]
QO_west_	G → T position +1 of intron 2 splice donor substitution. ΔGly164- Lys191. Aberrant mRNA splicing, intracellular degradation and no detectable protein	Emphysema reported in a compound heterozygote	[34]

Although other previously described AAT null alleles have been reported in Spain [10], now we describe the first new AAT null allele in Spain, in a Caucasian family from Madrid, designated as QO_Madrid_, as this city was the place of birth and residence of the index-case, as well as his three siblings and parents. These family cases showed a combination of two rare splicing variants in the donor splice site of intron 1C, QO_Porto_ and the new allele QO_Madrid_. Molecular characterization of these mutations located in regulatory region of the gene, allowed as to better understand the mechanisms of transcription and alternative splicing of *SERPINA1* gene.

## Methods

### Patients

**Figure 1:**
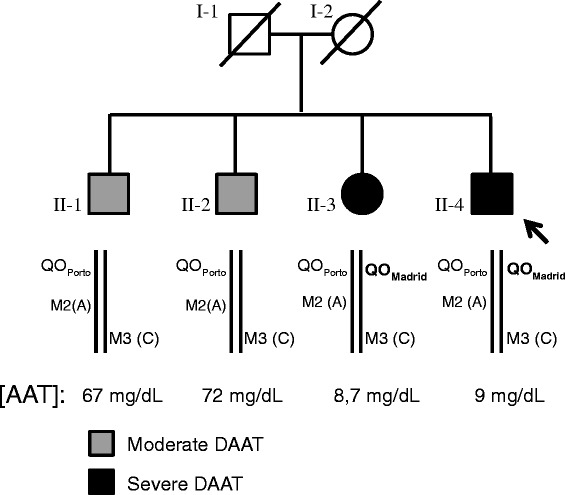
**Pedigree of the family studied.** Both parents (I-1 and I-2) were dead when the AAT study was performed. The four siblings studied correspond to 3 males (II-1, II-2 and II-4) and a female (II-3). The index case is II-4, indicated by the arrow. None of them had any children. Allele combination of the QO_Porto_ and QO_Madrid_ mutations and the normal variants, M2 (G/A, Arg101His) and M3 (A/C, Glu376Asp) found in each individual are depicted below each family member. Corresponding AAT serum level are also shown.

The family was composed by four siblings, three males and one female, two of them PI*QO_Porto_/QO_Madrid_ compound heterozygotes with severe AAT deficiency, and the remaining ones PI*M/QO_Porto_ as well heterozygotes but with moderate AAT deficiency (Figure 1). Their parents died years ago, and none of them had descendants or other blood relatives. Signed informed consent for the study was obtained from patients.

All these patients were previously characterized for AAT serum levels, AAT protein phenotype and genotyping of the common PI*S and PI*Z alleles. The determination of serum AAT was conducted by immunonephelometry with an autoanalyzer ArrayTM Protein System (Beckman-Instruments, Brea, California, USA).

Protease inhibitor (PI) typing was made by means of isoelectric focusing (IEF) technique as previously described [10,35]. All four siblings were studied for the existence of AAT protein variants by IEF but conclusive patterns were not found.

In addition, all subjects were initially genotyped for the commonest defective variants PI*Z and PI*S by a LightCycler PCR, using primers and hybridization probes as described before [36]. None of these common deficient variants were found in our cases.

### Sequence analysis of the entire *SERPINA1* gene

Since the low serum AAT concentration of the subjects could not be attributed to PI*Z genotypes, the entire coding sequence of the *SERPINA1* gene was analyzed (Reference sequences NG_008290.1, NM_000295.4). Exons 2 to 5 and exon-intron junctions were analyzed by means of a Sanger automated sequencing, using previously described primers [37]. After that, to exclude the existence of other rare variants outside coding regions of the gene, primers for amplification of additional gene fragments to cover the whole gene were designed (Additional file 1: Table S1). The entire gene sequence were analyzed by Sanger automated sequencing (ABI PRISM 377 Applied BioSystems) in the four studied individuals of the family.

### Expression analysis by RT-PCR

RNA extraction from peripheral blood was performed using RNAeasy kit (Quiagen) following manufacturer’s recommendations. Then, cDNA synthesis was carried out by reverse transcription PCR (RT-PCR) using the Maxima First Strand cDNA Synthesis kit (Thermo Scientific). To analyze transcripts and splicing (from normal hepatocytes and peripheral blood samples) occurring in patients and controls, the regions between E1A/E2, E1B/E2 and E1C/E2 or E1C/E5 were amplified using several primers located in exon 1A, 1B, 1C, exon 2 and exon 5 as follows: E1A-F: 5′-TCCTGTGCCTGCCAGAAGAG-3′; E1B-F, 5′-ATCAGGCATTTTGGGGTGACT-3′ [29]; E1C, 5′CTGTCTCCTCAGCTTCAGGC3′; E2-R, 5′-TTCTTGGCCTCTTCGGTGTC-3′ and E5-R, 5′-CCATGAAGAGGGGAGACTTGG-3′. In addition, a primer localized in the initial region of intron 1C INT1C, 5′-GGGGATGGAGAATGTGAGCC-3′ was also used to analyze the existence of possible transcripts using intron sequence in mutant cases. PCR amplification of the different products was performed under the following conditions: 35 cycles of 94°C for 45 s, 60°C for 45 s, and 72°C for 45 s. Amplified products were visualized in 1-2% agarose gels, purified by PCR purification Kit (Qiagen) and subsequently cloned into pGEM-T easy vector (Promega, Madison WI, USA) or directly analyzed by sequencing with an ABI PRISM 377 sequencer. Ligation reactions were used to transform DH5α competent cells. Clones containing PCR products were selected by blue/white colony and standard ampicillin selection. Positive transformants were analyzed by PCR and sequenced using AAT primers.

### Computational predictions

By using different prediction methods integrated in Alamut 1.3 software we evaluated splice signal detection (SpliceSiteFinder-like, MaxEntScan, GeneSplicer, Human Splicing Finder or Known constitutive signals) and Exonic Splicing Enhancers ESE binding site detection (ESEFinder) comparing reference and mutated sequences.

### Construction of the Minigene MGserp1a_ex1b-1c

In order to analyze the effect of the splicing variants, a minigene containing exons 1B and 1C with the flanking intronic sequences were constructed. Exons 1B and 1C and part of the flanking intronic sequences (1,252 bp; Additional file 2: Figure S2) were amplified with Phusion High Fidelity polymerase (Fisher Scientific, Madrid, Spain) and forward, 5′ *GCTCTAGAACTAGTGGATCCCCCGG*GGAGCAAAAACAGAAACAGG 3′ and reverse, 5′ *ATAAGCTTGATATCGAATTCCTGCA*CTTTGTTGCTGTTGCTGTATC 3′ primers (cloning tails are in italics). This fragment was cloned into the pSAD® splicing vector (patent# P201231427, Consejo Superior de Investigaciones Científicas, Spain) by overlap extension PCR [38], transformed into the DH5α strain of Escherichia coli (Life Technologies, Carslbad, CA, USA), and plated on LB-agar with ampicillin (Fisher Scientific) at 100 μg/μL, X-Gal (5-bromo-4-chloro-3-indolyl-beta-D-galactopyranoside, Fisher Scientific) at 40 μg/μL and IPTG 0.1 mM (isopropyl beta-D-thiogalactopyranoside, Fisher Scientific), where recombinant white colonies were selected. This construct constituted the minigene MGserp1a_ex1b-1c.

### In vitro expression analysis of splicing variants QO_Porto_ and QO_Madrid_ by site directed mutagenesis of the Minigene MGserp1a_ex1b-1c

To include in the minigene the studied genetic variants, mutagenesis was carried out according to PCR mutagenesis protocol over the wild type minigene by using Pfu UltraHF polymerase (Agilent, Santa Clara, CA) and primers for variant c.-5 + 1G > A (QO_Porto_) (F:5′ACTGACCTGGGACAGTGAATCATAAGTATGCCTTTCACTGCGA3′, R: 5′TCGCAGTGAAAGGCATACTTATGATTCACTGTCCCAGGTCAGT3′) and for variant QO_Madrid_, c.-5 + 2dupT (F:5′CTGACCTGGGACAGTGAATCGTTAAGTATGCCTTTCACTGCGAGA3′, and R:5′TCTCGCAGTGAAAGGCATACTTAACGATTCACTGTCCCAGGTCAG3′).

For transfection, approximately 10^5^ HeLa (human cervical carcinoma) cells were grown to 90% confluency in 0.5 mL of medium (DMEM, 10% Fetal Bovine Serum, 1% glucose and 1% Penicillin/Streptomycin; Fisher Scientific) in 4-well plates (Nunc, Roskilde, Denmark). Cells were transfected with 1 μg of each minigene and 2 μL of Lipofectamine 2000 (Life Technologies). To inhibit nonsense mediated decay (NMD), cells were incubated with cycloheximide (Sigma-Aldrich, St. Louis, MO) 300 μg/mL (4 hours).

### To analyze the expression products generated by the insertion of the variants, firstly

RNA from transfected cells was purified with the GeneMATRIX Universal RNA purification kit (EURx, Gdansk, Poland) with DNAse I treatment. Then, retrotranscription was carried out with 400 ng of RNA and the Transcriptor first strand cDNA synthesis kit (Roche Applied Science, Penzberg, Germany) and sequence specific primer SA2-PSPL3_RTREV (5′TGAGGAGTGAATTGGTCGAA3′) that retrotranscribes only RNA produced by the minigene. Two to five μL of cDNA were amplified with Platinum Taq polymerase (Life Technologies) and specific RT-PCR primers of the vector exons, SD6-PSPL3_RTFW (5′-TCACCTGGACAACCTCAAAG-3′) and SA2-PSPL3_RTREV. Samples were denatured at 94°C for 5 min, followed by 35 cycles consisting of 94°C for 30 sec, 59°C for 15 sec, and 72°C (1 min/kb), and a final extension step at 72°C for 5 min.

Semiquantitative fluorescent RT-PCRs were done with FAM-labelled SD6-PSPL3_RTFW primer as previously reported [39]. One μL of a dilution 1/10-1/20 of the RT-PCR products was mixed with 18.5 μL of Hi-Di Formamide (Life Technologies) and 0.5 μL of Genescan 500 Rox Size Standard (Life Technologies). To visualize the expression products, samples were run on an ABI3130 sequencer and analyzed with Peak Scanner (Life Technologies).

## Results

### Clinical and phenotypic characteristics of patients

The PI*QO_Porto_/QO_Madrid_ index case (II-4) was a male former smoker of 27 pack-years, diagnosed with severe COPD at age of 44, and currently receiving home oxygen therapy. He had dyspnea on moderate and small exertion, and radiological findings of diffuse panlobular emphysema and isolated bronchiectasis. Liver function tests were normal. He presented no detectable IEF bands and his serum AAT levels ranged from 9 to 17 mg/dL (Figure 1).

The other PI*QO_Porto_/QO_Madrid_ sibling (case II-3) was a never smoker asymptomatic female that maintained a nearly normal respiratory function, but early functional signs of lung damage ((Transfer coefficient (diffusion constant) of CO diffusion (KCO) slightly decreased), and mild signs of lung emphysema, detected by computed tomography (CT) scan at the age of 41. She also showed absence of IEF bands, and AAT serum levels between 8 and 15 mg/dL. Her liver function tests were within normal parameters.

The remaining two siblings (II-1 and II-2) aged 51 and 55, with PI*M/QO_Porto_ genotypes had a moderate AAT deficiency, with AAT serum values around 70 mg/dL, and IEF revealing a normal electrophoretic pattern, compatible with an M phenotype. This partial reduction in the AAT levels but a normal protein phenotype corresponds to their heterozygous genotype, constituted by one normal (M) and one null allele (QO_Porto_). These subjects suffered from a moderate congenital mental retardation of unknown etiology. None of them showed symptoms or signs of respiratory disease, and their lung function tests remain normal at present, despite both are former smokers (smoking exposure of 10 pack-years each one). The CT scan detected mild basal emphysema in II-1 and no pathological findings in II-2.

### Identification of the QO_Madrid_ mutation

**Figure 2:**
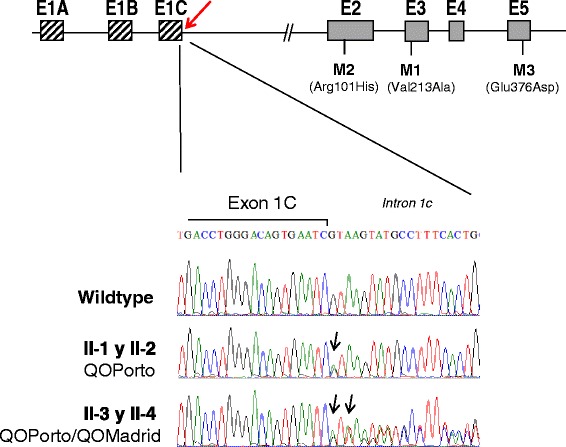
**Results of direct sequencing of the exon 1C-inton 1C boundary region.** Schematic representation of SERPINA1 gene is represented in the top showing position of the non-coding exons 1A, 1B and 1C and coding exons E2 to E5. Location of the common polymorphisms M1 (exon 3), M2 (exon 2) and M3 (exon 5) of this gene are also displayed. Position of the variants QO_Porto_ and QO_Madrid_ is marked with an arrow. Sequencing results of the patients are shown below. Comparison between a reference sequence from a normal individual (top sequence) and sequence from individuals II-1 and II-2 (middle panel) showing a heterozygous G to A change corresponding to the QO_Porto_. Bottom panel corresponds to direct sequencing of cases II-3 and II-4 that reveals heterozygosity for both QO_Porto_ and the new QO_Madrid_ (+2dupT) mutation.

The entire sequence of the *SERPINA1* gene, including coding and non-coding regions, was analyzed. DNA sequencing of the exon 1C and intron 1C revealed in the four siblings a heterozygous base substitution G to A at position +1 of the splice donor site of intron 1C (NM_000295.4:c.-5 + 1G > A), previously described as QO_Porto_ splicing mutation [29]. Analysis of the complete sequence of the *SERPINA1* gene showed that this was the only pathogenic mutation found in individuals II-1 and II-2 (Figure 2). The heterozygous status for QO_Porto_ allele in these patients was compatible with their moderate AAT deficiency.

Interestingly, the other two family members, II-3 and II-4, who showed a severe AAT deficiency, in addition to the QO_Porto_ mutation were carriers of a duplication of the thymine (T) in position +2 of the same splice donor site of intron 1C (NM_000295.4: c.-5 + 2dupT) (Figure 2). Since splicing sites are highly conserved sequences, this variation presumably disrupts the splicing donor site of this intron. This effect would be compatible with the strong AAT deficiency that both subjects presented. Since the whole sequence of the gene, coding and noncoding regions, was analyzed in these two patients, and there was no other pathogenic change, we assumed reasonably that this change is a deficient allele, not previously described, that was termed QO_Madrid_ following the classical nomenclature agreed by experts in the field, with QO representing a non-expressing gene at the protein level, and Madrid the name of the place of origin of the first carrier of the allele [40,41].

In addition, all four patients were heterozygous for the M2 (G/A, Arg101His, rs709932) and M3 (A/C, Glu376Asp, rs1303) common polymorphisms of the *SERPINA1* gene. Sequencing of the expression products generated by using primers located in exon 1C and exon 5 demonstrated that patients II-1 and II-2, carriers of the QO_Porto_ allele, only expressed transcripts with the M3 (C, Asp376) variant. Since QO_Porto_ mutation affects the normal expression of AAT mRNA, QO_Porto_ mutation in these patients occur in the other not expressed allele carrying the M2 (A, His101) but not the M3 (C, Asp376) variant (Figure 1). Consequently, the QO_Madrid_ mutation found in patients II-3 and II-4 segregated with the M3 (C, Asp376) variant.

### QO_Madrid_ impairs normal splicing in *in silico* analysis

**Figure 3:**
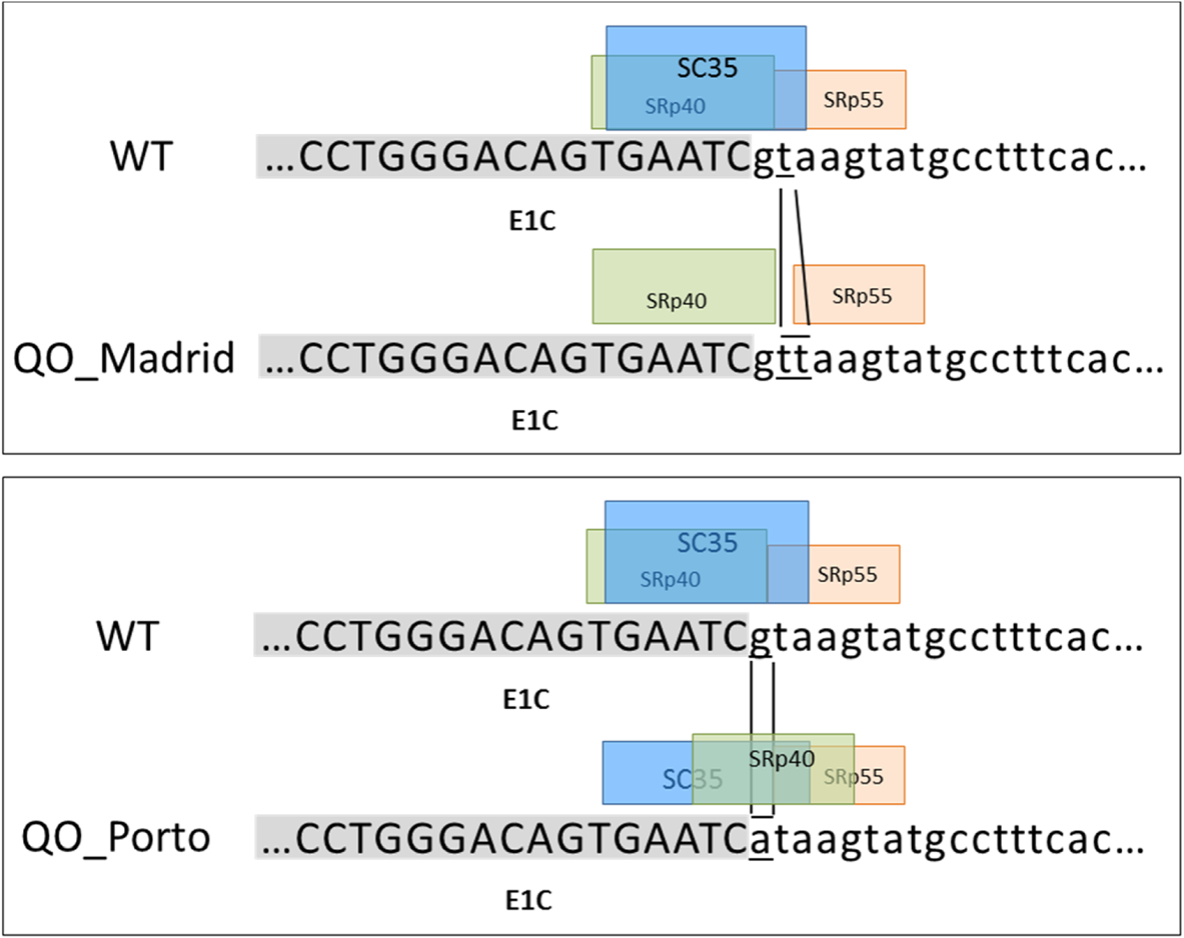
**Schematic representation of the region E1C-Intron 1C containing the mutations QO**_**Porto**_ **and QO**_**Madrid**_ **.** The consensus donor splicing sequence is disrupted in both the QO_Porto_ mutation and the QO_Madrid_ mutation. Boxes represent the scores of splicing factors obtained by bioinformatic tools. Several putative binding sites for splicing enhancer elements, SC35, SRp55 or SRp40, were predicted to be affected by these mutations. In the top panel, duplication of T of the QO_Madrid_ variant cause that site for SC35 disappear, and in the bottom panel the QO_Porto_ variant cause a reduction of the score value for the SC35 from 3.48 in the reference sequence to 3.12 in the mutated sequence. WT: wild type sequence.

Thymine duplication at the site of splicing of intron 1C (i.e., the QO_Madrid_ mutation) is located in a highly conserved splicing sequence of vertebrate splice donor sites and may lead to deregulation of gene expression. Computational predictions of splicing signals demonstrated disappearance of the constitutive donor splicing signals in the case of the QO_Madrid_ mutation, similarly to what happened for the QO_Porto_ splicing mutation. In addition several putative binding sites for Exonic Splicing Enhancers (ESE elements), such as SC35, SRp55 or SRp40 splicing factors, were predicted to be also affected by the duplication of T in position +2 of the QO_Madrid_ allele. Thus, SC35 site would be disrupted by QO_Madrid_ mutation and the predicted scores for SRp55 or SRp40 were reduced (Figure 3).

### Expression analysis of severe AAT deficiency cases carrying QO_Porto_ and QO_Madrid_

**Figure 4:**
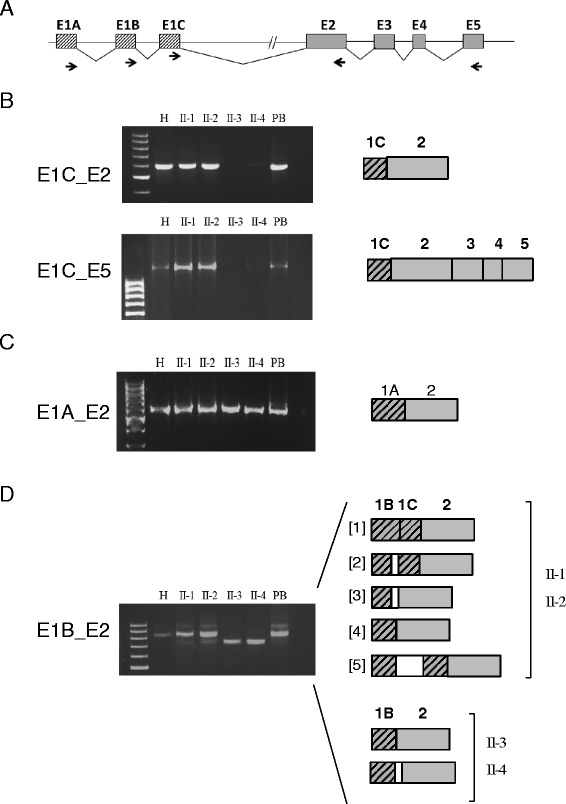
**Expression analysis in the family patients carrying the QO**_**Porto**_ **mutation (II-1 and II-2) and the ones carrying both QO**_**Porto**_ **and QO**_**Madrid**_ **variants (II-3 and II-4). A)** Schematic representation of the *SERPINA1* gene. To amplify different transcripts some forward primers in exons 1A, 1B, and 1C and reverse primers in exons 2 and 5 were designed (arrows). **B)** RT-PCR amplification of mRNA using primers located in exon 1C and the reverse primers in exon 2 or exon 5, analyzed in normal hepatocytes (H), the AAT cases, and in a normal peripheral blood sample (PB). No expression products were found in cases II-3 and II-4 when using exon 1C primer. All the other cases showed a single band of 587 bp corresponding to expression products containing the exon 1C directly spliced to exon 2, or in the case of E1C-E5 the expression product corresponded to a fragment of 1318 bp with the exon 1C joined to all the coding exons (2 to 5). **C)** Fragments generated by amplification of expression products using primers in exon 1A and exon 2. All cases showed expression of a transcript including the exon 1A directly joined to exon 2. No other alternative splicing variants were found. **D)** Expression analysis using primers in exon 1B revealed multiple bands meaning that alternative splicing occurred between exons 1B and 1C. After cloning, we differentiated five different splicing forms, some showed the use of alternative splicing sites on exon 1B (3 and 4) previously described. One of the transcription species retained the intron 1B (5). Severe DAAT cases only express transcripts without exon 1C.

To further investigate the molecular effect on splicing and expression of the *SERPINA1* gene with the QO_Madrid_ mutation which co-occurred with QO_Porto_ mutation in patients II-3 and II-4, expression products generated by different primer pairs in exons 1A, 1B, 1C and exons 2 and 5 were analyzed (Figure 4A). RT-PCR expression analysis of fragment E1C-E2 showed normally spliced products in control liver tissue and peripheral blood, as well as in the two moderate AAT deficiency patients II-1 and II-2 with PI*M/QO_Porto_ genotype (Figure 4B). In these patients sequence analysis demonstrated only one expressed allele based on the Arg101His polymorphism, corresponding to the normal M3 allele. However, no expression products were detected in patients carrying both splicing variants QO_Porto_ and QO_Madrid_, clearly indicating that QO_Madrid_ mutation also impairs normal splicing of intron 1C and represents a new null allele of the *SERPINA1* gene.

We also checked using primers in the E1C-Int1C whether transcripts including intron 1C sequence were expressed because of disruption of the splicing site due to the presence of both intron 1C splicing mutations, QO_Porto_ and QO_Madrid_. Similar to what was described for the QO_Porto_ mutation [29], no expression products retaining intron 1C sequence were detected indicating that if these transcripts are synthesized they might be probably rapidly degraded.

Transcripts that include E1A and E1B were also analyzed. Amplification of the E1A/E2 fragment in all patients, normal peripheral blood samples, and in a normal liver sample analyzed, produced one single band visible in agarose gel in all cases. Sequencing analysis revealed that the only mRNA product resulted from the direct splice of exon 1A to exon 2 (Figure 4C). Alternative spliced forms, previously described by others in monocytes/macrophages [8,42], were not detected in our samples. Moreover, in all patients transcripts which include E1A showed expression of both alleles, since both variants Arg101 and His101 (G/A) were found. This indicates that QO_Porto_ and QO_Madrid_ mutations do not impede transcription not requiring the splice donor site of intron 1C.

Regarding transcripts which included exon 1B, multiple expression products were detected after amplification with primer pair E1B-E2. After cloning, five different products were identified in patients II-1 and II-2 (Figure 4D). The most frequent species, as shown by the number of colonies, were those including exons 1B joined to 1C and to exon 2 (named as product [1] in Figure 4D). Alternative products ([2] and [3] in Figure 4D) generated using a cryptic splice donor site in exon 1B previously described, producing a 18 bp shorter fragment at the 3′ end of the exon 1B, were also found [43]. In addition, alternatively spliced products formed by direct splice of exon 1B to E2 ([4] in Figure 4D), as well as transcripts retaining the intron 1B ([5] in Figure 4D), were also found but in less amount.

In patients with QO_Porto_ and QO_Madrid_ mutations (II-3 and II-4) only two different transcripts species were detected by using E1B and E2 primer pair. In these cases both fragments corresponded to RNA products containing only E1B joined to E2 (Figure 4D), one of the products previously described using the cryptic splice donor site in exon 1B [44]. However, these patients do not include E1C in transcription products since splice donor site of intron 1C is completely disrupted by the presence of QO_Porto_ and QO_Madrid_ splicing mutations.

### Functional assays by hybrid minigenes

**Figure 5:**
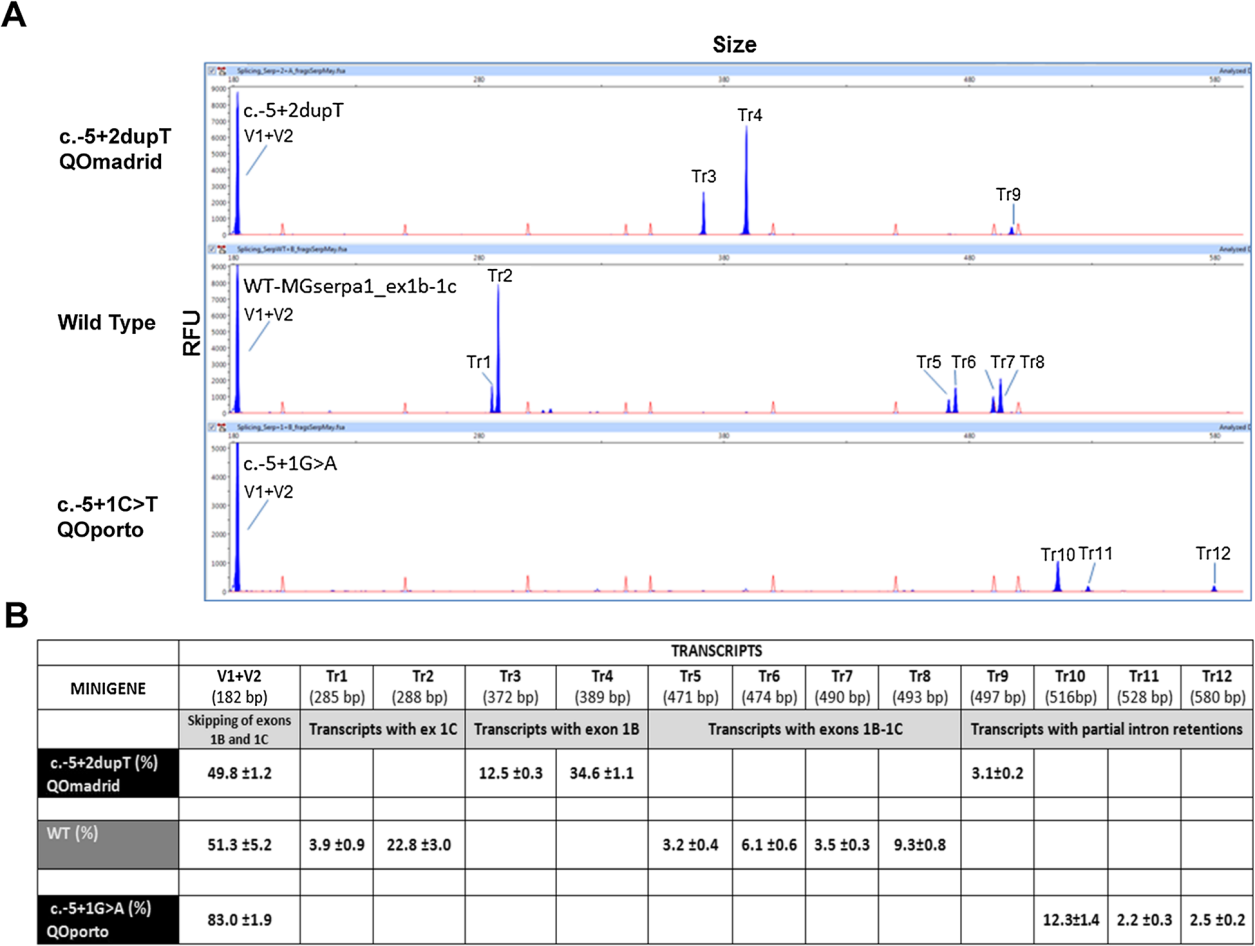
**Transcripts detection in wt and splicing mutation minigenes. A)** Fluorescent capillary electrophoresis of RT-PCR products generated by the wild type, c.-5 + 2dupT and c.-5 + 1G > A minigenes. Screenshots of Peak Scanner electropherograms are shown. Fluorescent RT-PCRs (blue peaks) of wt and mutant minigenes were run in an ABI3130 DNA sequencer with Genescan ROX 500 (red peaks) as size standard. RFU: Relative Fluorescence Units. **B)** Quantification of all detected of transcripts (Tr1 to 12) generated by the wild type, or mutants c.-5 + 1G > A (QO_Porto_) and c.-5 + 2dupT (QO_Madrid_) minigenes of the *SERPINA1* gene are represented with the mean proportion of each one. Sizes were calculated by the Peak Scanner software. Depending the use of the alternative splicing sites described for exon 1B and 1C, the deduced transcript composition is: Tr1: V1-1Cs -V2; Tr2: V1-1Cl -V2; Tr3: V1-1Bs -V2; Tr4: V1-1Bl -V2; Tr5: V1-1Bs -1Cs-V2; Tr6: V1-1Bs -1Cl-V2; Tr7: V1-1Bl -1Cs-V2; Tr8: V1-1Bl -1Cl-V2; Tr9-12: partial intron retentions. (1Bs and 1Bl: exon1B short and long, respectively; 1Cs and 1Cl: exon 1C short and long, respectively).

The wild type (wt) and the mutant minigenes were functionally assayed in HeLa cells. The wt construct produced at least 7 different transcript as well as a wide range of rare splicing isoforms (Figure 5), where the skipping of both exons 1B and 1C (only with vector exons v1 + v2) was the most frequent event (51.3%). This isoform resembles the most abundant transcript of AAT in macrophages without exons 1B and 1C [43]. We also identified four distinct isoforms with exons 1B and 1C that resulted from the combination of four splice sites: the canonical donor and acceptor sites, and one donor (18 nucleotides downstream in intron 1B) and one acceptor (3 nucleotides downstream in exon 1C) cryptic sites (transcripts Tr5, Tr6, Tr7 and Tr8, together accounting for 22.1%; Figure 5). Finally, Tr1 and Tr2 would represent transcripts lacking exon 1B. These results are in accordance to the transcripts described by Rollini and Fournier (2000) [43]. Therefore, the splicing pattern of wild type MGserp1a_ex1b-1c mimicked that of macrophages cell lines.

Interestingly, mutations c.-5 + 1G > A and c.-5 + 2dupT triggered the elimination of six transcripts of the wild type minigene (Tr1, 2, 5, 6, 7, 8) and generated six new ones, Tr10, Tr11 and Tr12 (c.-5 + 1G > A), and Tr3, Tr4 and Tr9 (c.-5 + 2dupT). The V1 + V2 transcript was significantly enriched in c.-5 + 1G > A (83.0% vs. 51.3% in wt) although other three aberrant transcripts (Tr10-12) would correspond to partial retentions of intron 1C. The new mutation QO_Madrid_ (c.-5 + 2dupT) induced isoforms Tr3 and 4 that were relatively abundant (12.5 and 34.6%, respectively) and corresponded to exon 1C skipping and alternative usage of the two donor sites of intron 1B. This result reproduced the splicing profile of patient RNA.

## Discussion

We have characterized a new splicing variant in the *SERPINA1* gene producing a null allele that we named PI*QO_Madrid_ following the traditional terms used to designate the new genetic variants found in this condition. This new allele increases the short list of null alleles reported to date, which are characterized by the almost absence serum AAT [4].

As shown in Table 1, several molecular mechanisms have been described to be responsible for the lack of expression of AAT protein including deletion of AAT coding exons, nonsense mutations producing formation of premature stop codons or splicing mutations. The QO_Madrid_ variant corresponded to the fourth splice site alteration described so far in the *SERPINA1* gene. The three other reported splicing variants were QO_West_, QO_Bonny blue_ and QO_Porto_. The QO_West_ and QO_Bonny blue_ alleles are caused by alterations in the splice donor and acceptor sites of intron 2 [4,34], while QO_Porto_ corresponded to a G to A transition in the donor splice site of intron 1C [29]. Therefore, the QO_Madrid_ adds a new splicing variant (+2dupT) to the same splicing donor site of intron 1C.

This new QO_Madrid_ mutation, which occurred in a M3 background, enlarge the number of pathogenic mutations outside the coding sequence of the *SERPINA1* gene and highlight the importance that regulatory regions might have in the disease. Studies of these regions should be seriously considered in discordant cases with severe AAT deficiency where no coding alterations are found.

In the two severe AAT deficiency patients of this family in Madrid, the new splicing mutation occurred in heterozygous state in combination with another splicing mutation previously described as QO_Porto_, curiously both affecting the donor splice site of intron 1C. Both composite heterozygotes PI*QO_Porto_/QO_Madrid_ individuals showed extremely low levels of serum AAT and a practically total absence of detectable plasma protein by isoelectric focusing, indicating that these changes strongly affect the AAT synthesis by disrupting the normal splicing of the intron 1C. In this regard, we demonstrate that these patients lacked of transcripts with exon 1C, and therefore of the properly matured mRNA synthesized by the liver which is the major source of the AAT mRNA. Nevertheless, these two composite heterozygotes were able to produce AAT mRNA transcripts from exons 1A and 1B, which are probably originated from the macrophage-specific promoter localized in the gene upstream of the liver promoter [8]. Is it possible then, that the low level of serum AAT protein detected in severe patients was produced by macrophages or other cells than hepatocytes. However, these transcripts represent a small proportion of the total AAT transcription, and may be insufficient to compensate the negative effects of the splicing mutations in liver cells.

In the index case, the presence of both QO_Porto_ and QO_Madrid_ mutations likely caused the genetic susceptibility to early onset emphysema after smoking exposure, similarly to which occur with other null alleles [27]. The siblings presenting a genotype PI*M/QO_Porto_ showed AAT serum levels concordant with the presence of a normal and a mutated allele. Heterozygous status for a null allele is generally considered to produce enough normal protein to keep enough antielastase activity in the lung, and hence the risk of lung disease might be mild. These patients also presented moderate degree of mental retardation. Dementia-like syndrome associated with serpinopaties has been reported [9] but there is no evidence suggesting that QO_Porto_ could be related with mental impairment detected in these individuals. Although splicing variants have been rarely described causing AAT deficiency, their existence show that correct splicing events are important for the accurate function of this gene. Splicing alterations have been described to originate genetic diseases such as spinal muscular atrophy, neurofibromatosis type I or myotonic dystrophy, among many others. Numerous disease causing genes undergo alternative splicing to regulate expression, and it is estimated that overall 15% of mutations are located within splicing sites and more than 20% of missense mutations lie within predicted splicing elements [45].

Computational analysis of the effect of the new QO_Madrid_ revealed the disruption of the consensus donor splicing site sequence. In addition, using exonic splicing enhancers (ESE) motif predictions it was observed that the variant mainly eliminate a potential SC35 factor binding site. This and other ESE sequences have been described to play a key role in regulating splicing events [46].

Transcriptional analysis of the AAT deficiency family members allowed us to verify that transcription from exon 1C, still detected in siblings with PI*M/QO_Porto_, was totally impaired in the compound heterozygotes PI*QO_Porto_/QO_Madrid_. The QO_Madrid_ mutation highlights the importance of exon 1C for the correct expression of AAT in the liver, in order to ensure the production of appropriate serum AAT concentrations. Even though transcripts from the exons 1A and 1B were detected in the compound heterozygotes, these RNA species might not be abundant enough to provide acceptable AAT serum levels.

Moreover, we have constructed and validated the minigene MGserpa1_ex1b-1c in the pSAD vector where the splicing patterns of mutant and wt minigenes were clearly distinguished. Consequently, it constitutes a very valuable tool for the functional and clinical classification of DNA variants from disease genes, facilitating the discrimination between neutral and deleterious changes. This approach also allows quantifying the impact on splicing of a single variant without the interference of another allele as it occurs in patient RNA. Moreover, at least 13 different transcripts from the AAT construct were detected supporting the high sensitivity and resolution of fluorescent capillary electrophoresis of RT-PCR products as previously reported [39]. Furthermore, the wild type construct and several cell lines displayed similar splicing patterns [43] and variant c.-5 + 2dupT in minigene and patient RNA was associated with exon 1C skipping in both cases, lending further support to the reproducibility of minigene assays. Indeed, all the transcripts detected in macrophage cell lines were identified in our wild type minigene, including the alternative selection of cryptic splice sites in intron 1B and exon 1C. 3.

## Conclusion

In summary, a new variant in the 5′UTR promoter region of the *SERPINA1* gene causing a new null allele QO_Madrid_ has been described for a first time. This new variant is added to the previously described null variants that affect splicing causing the disease. The minigene system is a powerful approach to detect variants with an impact on splicing and have contributed to a better knowledge of this gene expression step by quantification of normal and aberrant alternative transcripts of the *SERPINA1* gene.

Similar to what occur with deficient variants located in exons, which allowed a better understanding of the AAT structure-function relationships, splicing variants provide us with more insight into the expression regulation of the *SERPINA1* gene.

## Additional files

Additional file 1: Table S1. List of primers used to amplify all fragments of the entire SERPINA1 gene (Ensembl: ENSG00000197249). Additional file 2: Figure S1. Scheme of minigene MGserpa1_ex1b-1c.

## Abbreviations

AAT: Alpha-1 antitrypsin
QO: Null allele
IEF: Isoelectric focusing
ESE: Exonic Splicing Enhancers
CT: Computed tomography
