# Supplementary material for: Severe alpha-1 antitrypsin deficiency in composite heterozygotes inheriting a new splicing mutation QOMadrid
Source: Respir Res. 2014 Oct 7;15(1):125. doi: 10.1186/s12931-014-0125-y (PMC4194419; doi:10.1186/s12931-014-0125-y)
Supplement: Additional file 1: Table S1. — List of primers used to amplify all fragments of the entire SERPINA1 gene (Ensembl: ENSG00000197249). [file 12931_2014_125_MOESM1_ESM.docx]

**Additional Table.**

**List of primers used to amplify all fragments of the entire SERPINA1 gene (Ensembl: ENSG00000197249)**

| **Fragment** | **Forward primer (5’-3’)** | **Reverse primer(5’-3’)** | **Product Size (bp)** |
| --- | --- | --- | --- |
| F1 | TGGTGCGTTTTTCCAGATTA | GCACAGTGCCCAGTTCCT | 624 |
| Fex1A | GCCTCCACCCGAAGTCTAC | GTGGAACTGAGTGAGCAGCA | 316 |
| F2 | GCTTGAGGAGAGCTTGAGGA | CTTGTTGAGCTGCTGTGAGG | 554 |
| F3 | CACACCCAGTACCCGTCTCT | TGGGTTAGAGCCCATTTGAC | 978 |
| F4 | GGCTGACTGGGTAACTTTGG | ATGCCCAAGCAGTAGGAGAG | 979 |
| F5 | TGCACAGCTCCTCTGTCTGT | AAAGGTGGACTTTGTCATAGAGC | 922 |
| F6 | TGCAGCAATAGCAGCAAGAA | CGCCTATGGTCAAACAACCT | 983 |
| F7 | CCTGGTCACACTTGGGTTTA | CACGCCTGGCTAATTTTTGT | 986 |
| F8 | CGGATCAAGAGGTCAGGAGT | TGGGAAAACTGAGGTCCTAGC | 931 |
| F9 | CTGCCGGGCATATTCTCCT | ATGGAGGGGCTGTGATTAAA | 807 |
| F10 | GAGGATCCTTGTGAGTGTTGG | GTCCTGCAAGACAGAGATGG | 698 |
| Fex2 | ACGTGGTGTCAATCCCTGATCACTG | TATGGGAACAGCTCAGGCTGG | 764 |
| F11 | CACAGTTTTTGCTCTGGTGAA | GAGCCTCAGAGTGGAGGAAG | 777 |
| F12 | TCCCAGGAAAACAGCATAGG | TCCTCGGTGTCCTTGACTTC | 823 |
| Fex3 | CTTCCAAACCTTCACTCACCCCTGGT | GTCCTCATGGAGCATGGACGGCG | 520 |
| F13 | ATGAGGCCACCACTGAGTTC | GCCCAGTGACAACCGTTTAG | 757 |
| F14 | AAAACCAGAGAGGAGCACTCA | AGGCATGGCACAGCATTC | 700 |
| Fex4 | CACTTGCACTGTGGTGGGTCCCAG | TTCTTCCCTACAGATACCAGGG | 273 |
| F15 | CCTTGTTGCACCCTGGTATC | TCAGTCCCTTTCTCGTCGAT | 847 |
| Fex5 | GAGCCTTGCTCGAGGCCTGGGATC | CAGAGAAAACATGGGAGGGATTTACA | 372 |
