# Supplementary figures and images for: Severe alpha-1 antitrypsin deficiency in composite heterozygotes inheriting a new splicing mutation QOMadrid
Source: Respir Res. 2014 Oct 7;15(1):125. doi: 10.1186/s12931-014-0125-y (PMC4194419; doi:10.1186/s12931-014-0125-y)

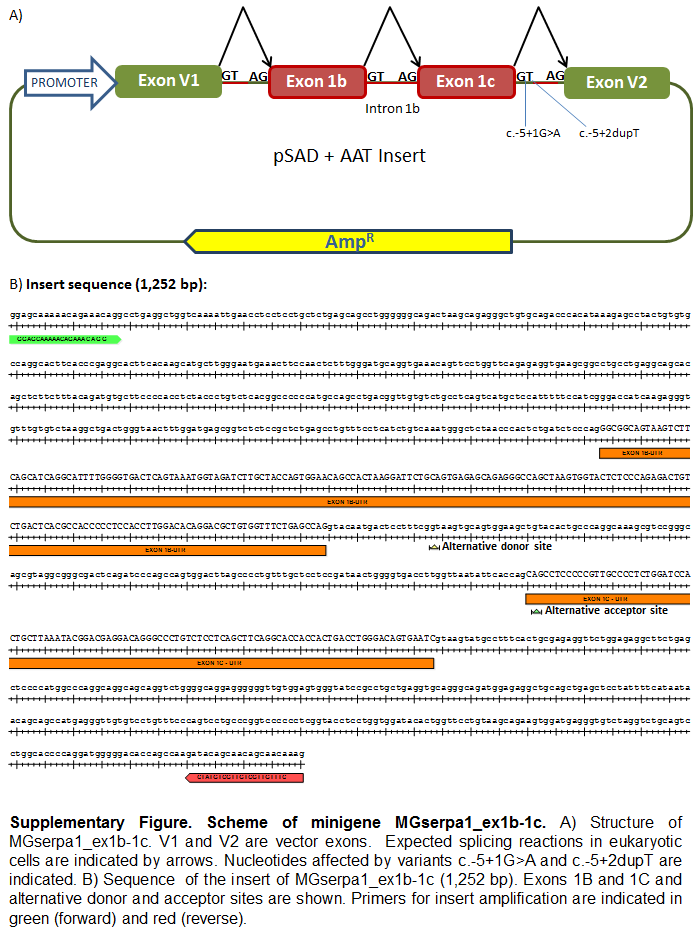

Supplement: Additional file 2: Figure S1. — Scheme of minigene MGserpa1_ex1b-1c. [file 12931_2014_125_MOESM2_ESM.tiff]
